# Tuberculosis patients in an Indian mega-city: Where do they live and where are they diagnosed?

**DOI:** 10.1371/journal.pone.0183240

**Published:** 2017-08-15

**Authors:** Ramnath Subbaraman, Beena E. Thomas, Senthil Sellappan, Chandra Suresh, Lavanya Jayabal, Savari Lincy, Agnes L. Raja, Allison McFall, Sunil Suhas Solomon, Kenneth H. Mayer, Soumya Swaminathan

**Affiliations:** 1 Division of Infectious Diseases, Brigham and Women’s Hospital and Harvard Medical School, Boston, Massachusetts, United States of America; 2 Department of Social and Behavioral Research, National Institute for Research in Tuberculosis, Chennai, India; 3 District Tuberculosis Office, Chennai, India; 4 Department of Epidemiology, Johns Hopkins University Bloomberg School of Public Health, Baltimore, Maryland, United States of America; 5 Division of Infectious Diseases, Beth Israel Deaconess Medical Center and Harvard Medical School, Boston, Massachusetts, United States of America; 6 Indian Council of Medical Research, New Delhi, India; Food and Drug Administration, UNITED STATES

## Abstract

**Objective:**

Tuberculosis (TB) is a major source of mortality in urban India, with many structural challenges to optimal care delivery. In the government TB program in Chennai, India’s fourth most populous city, there is a 49% gap between the official number of smear-positive TB patients diagnosed and the official number registered in TB treatment within the city in 2014. We hypothesize that this “urban registration gap” is partly due to rural patients temporarily visiting the city for diagnostic evaluation.

**Methods:**

We collected data for one month (May 2015) from 22 government designated microscopy centers (DMCs) in Chennai where 90% of smear-positive TB patients are diagnosed and coded patient addresses by location. We also analyzed the distribution of chest symptomatics (i.e., patients screened for TB because of pulmonary symptoms) and diagnosed smear-positive TB patients for all of Chennai’s 54 DMCs in 2014.

**Results:**

At 22 DMCs in May 2015, 565 of 3,543 (15.9%) chest symptomatics and 71 of 412 (17.2%) diagnosed smear-positive patients had an address outside of Chennai. At the city’s four high patient volume DMCs, 54 of 270 (20.0%) smear-positive patients lived out-of-city. At one of these high-volume DMCs, 31 of 59 (52.5%) smear-positive patients lived out-of-city. Out of 6,135 smear-positive patients diagnosed in Chennai in 2014, 3,498 (57%) were diagnosed at the four high-volume DMCs. The 32 DMCs with the lowest patient volume diagnosed 10% of all smear-positive patients.

**Conclusions:**

TB case detection in Chennai is centralized, with four high-volume DMCs making most diagnoses. One-sixth of patients are from outside the city, most of whom get evaluated at these high-volume DMCs. This calls for better coordination between high-volume city DMCs and rural TB units where many patients may take TB treatment. Patient mobility only partly explains Chennai’s urban registration gap, suggesting that pretreatment loss to follow-up of patients who live within the city may also be a major problem.

## Introduction: The urban tuberculosis registration gap

India has the world’s largest tuberculosis (TB) epidemic, with nearly one-quarter of the global burden of incident TB patients annually, a considerable proportion of whom are treated in the Government of India’s Revised National TB Control Programme (RNTCP) [1]. The RNTCP faces considerable challenges in retaining TB patients throughout the process of diagnostic workup, linkage to care, and treatment [2].

RNTCP statistics for cities in India indicate potential challenges around patient mobility and linkage to care. For India’s largest cities, with the exception of Pune, more patients are diagnosed with smear-positive TB every year than the number of smear-positive patients who are registered for TB treatment within those cities—a phenomenon we refer to as the “urban TB registration gap” (Table 1) [3]. Chennai, Bengaluru, and Hyderabad have the largest registration gaps; only about half of all diagnosed smear-positive TB patients are registered for treatment within these cities. Overall, about one-quarter of diagnosed smear-positive TB patients are not registered for treatment within these cities.

**Table 1:** The tuberculosis “urban registration gap” in major Indian cities, 2013.

City	Smear-positive TB patients diagnosed at RNTCP facilities within the city	Smear-positive TB patients registered for treatment at RNTCP facilities within the city	Urban Registration Gap (unaccounted for smear-positive TB patients) *N (%)*
Ahmedabad	6,113	4,384	1,729 (28.3%)
Bengaluru	6,150	2,934	3,216 (52.3%)
Chennai	6,135	3,148	2,987 (48.7%)
Delhi	22,345	19,182	3,163 (14.2%)
Hyderabad	5,701	2,836	2,865 (50.3%)
Kolkata	4,733	3,201	1,532 (32.4%)
Mumbai	14,269	11,699	2,570 (18.0%)
Pune	1,864	2,026	-162 (-8.7%)
Surat	2,492	1,556	936 (37.6%)
Total	69,726	50,966	18,760 (26.9%)

TB = tuberculosis; RNTCP = Revised National TB Control Programme. Statistics are from the 2014 RNTCP annual status report [3], and Chennai’s District TB Office.

What explains this large proportion of “missing” urban TB patients? We have two hypotheses for the urban TB registration gap. First, we hypothesize that this gap is partly due to pretreatment loss to follow-up (previously called “initial default”)—that is, that some patients diagnosed at TB designated microscopy centers (DMCs) subsequently do not start TB therapy or choose to take therapy in the private sector, where their subsequent status is usually not registered by the public health system.

Our second hypothesis is that the urban registration gap is partly explained by patients coming from rural areas who temporarily visit cities to access TB diagnostic services. This movement into cities for medical evaluation could be due to poor access to government health services in rural areas [4], patient perceptions that they will receive higher quality care in cities, or fear of loss of confidentiality and stigma when seeking care locally. After diagnosis, these patients may be referred back to rural areas to start TB treatment, thereby not showing up in city TB treatment statistics. If true, and if these patients are being successfully linked to care in rural areas, the urban registration gap may simply be explained by high patient mobility and may not be indicative of poor healthcare delivery. If a large proportion of patients seeking care in the city are from rural areas, this finding may have important implications for designing urban TB services in a manner that facilitates linkage to care.

The question of where TB patients in cities are coming from also intersects with an interrelated question of where TB patients are being diagnosed in these cities. In 2014, the Chennai RNTCP had 54 TB DMCs located at tertiary, secondary, and primary health facilities. Understanding the relative distribution of new TB diagnoses across these facilities—whether diagnoses tend to be centralized at a few high patient volume tertiary centers or widely distributed among numerous primary and secondary health centers—also has implications for strengthening urban TB services. With the emergence of higher-quality but relatively expensive diagnostic modalities, such as Cartridge Based Nucleic Acid Amplification Tests (CB-NAATs), understanding where most TB patients are being diagnosed may guide initial placement of these new diagnostic tests at specific centers, in order to have the biggest impact—at least until resources are available to scale-up CBNAATs more broadly to reach all patients who might benefit from these tests.

In this paper, we first analyze patient address data from DMCs in Chennai to understand where chest symptomatics (i.e., people with suspected TB) and newly diagnosed smear-positive TB patients are coming from. Second, we evaluate the pattern of where TB patients are being diagnosed in Chennai, to understand the relative importance of tertiary, secondary, and primary health centers for detecting TB patients.

## Methods

### Study settings

Chennai is India’s fourth largest city, with a population of 7 million people. RNTCP DMCs in Chennai evaluate 63,000 to 67,000 patients with sputum microscopy and diagnose 5,600 to 6,300 smear-positive cases annually [3, 5, 6]. Three-fourths of patients in the RNTCP have a household income of U.S. $2 (Indian Rupees 136) a day or less [7].

In 2014, there were 54 government DMCs in Chennai where patients could get evaluated for TB by sputum smear microscopy. As per RNTCP protocol, newly diagnosed TB patients are subsequently referred to one of 25 TB Units in the city, or to TB Units elsewhere in Tamil Nadu state, to start therapy. Each TB Unit consists of a cluster of directly observed therapy (DOT) centers in a specific locale, usually based out of the city’s primary health centers (PHCs). Patients are referred to the TB Unit closest to their homes for ease of travel to a DOT center to undergo observed dosing for TB medications three times a week.

### Analysis of where TB patients live

Based on the analysis of data from Chennai’s 54 DMCs, we found that >90% of smear-positive TB patients were diagnosed at just 22 of these DMCs. To understand where TB patients live, we therefore copied the complete patient registers for the month of May 2015 from these 22 DMCs. For purposes of study feasibility, we exclude the remaining 32 DMCs from the analysis, since together they accounted for <10% of the city’s smear-positive diagnoses.

From August to October 2015, research investigators visited each of the 22 DMCs across Chennai and collected data for all register entries, including patient address data, which were entered into a REDCAP database. For addresses within Chennai, the pin code was entered to be able to classify patient locations within general areas of the city. Addresses located outside of Chennai were classified based on the district in Tamil Nadu in which the town or village is located. Patient demographic data (age and gender), prior TB history, sputum smear results, line probe assay results, and HIV status (if known) were also entered into the database. The final de-identified dataset and data dictionary are available in the S1 Appendix and S2 Appendix, respectively.

Four of the 22 DMCs in Chennai for which we collected data diagnosed the majority of smear-positive patients in the city: Government Thiruvatteeswarar Hospital of Thoracic Medicine (also known as Otteri TB Hospital), the Institute of Thoracic Medicine, Government Stanley Hospital, and Chennai General Hospital (also known as Madras Medical College). We will hereafter refer to these four centers as the “high-volume DMCs”. We conducted a sub-analysis of address locations for chest symptomatics and smear-positive patients at these high-volume DMCs, given their disproportionate patient volume.

Using a dataset with de-identified patient information, JMP Pro 12 statistical software was used to generate descriptive statistics on the specific areas of Chennai or districts within Tamil Nadu where chest symptomatics (i.e., people with suspected TB who submit sputum specimens at DMCs) and diagnosed smear-positive patients (i.e., chest symptomatics who have at least one positive sputum smear) live. We excluded 336 patients who were already on TB therapy and who had provided sputum samples for follow-up evaluation and 153 patients who had no address listed, of whom 11 were diagnosed smear-positive patients. This left 3,543 patient entries in the dataset for analysis. We tested for significant differences in proportions using the Chi-squared test.

We used ArcGIS 10.3.1 software by Esri® to generate a heat map of Chennai based on the pincodes where chest symptomatics and smear-positive patients reside. Pincodes, or “postal index numbers”, are six-digit codes used by the Indian postal system to divide the country into different geographical administrative areas. We procured a commercially available pincode map from MBI International (Michael Bauer International GmbH, Michael Bauer Research GmbH, Germany, 2013) of Chennai, which has 298 distinct pincodes. An ArcGIS basemap was used for all maps (source: OpenStreetMap contributors, www.opendatacommons.org/licenses/odbl). The OpenStreetMap open-access license is available in the S3 Appendix.

### Analysis of where TB patients are evaluated and get diagnosed

De-identified datasets containing information on the numbers of TB patients diagnosed at DMCs and treated at TB Units were obtained from Chennai’s district TB office. We obtained data regarding the total number of chest symptomatics screened and total number of smear-positive patients diagnosed on a monthly basis at all 54 city DMCs in 2014. We tabulated these figures in a Microsoft Excel spreadsheet to estimate the proportion of chest symptomatics evaluated and the number of smear-positive TB patients diagnosed at each DMC in 2014.

### Analysis of where TB patients start treatment

We also obtained data from Chennai’s district TB office regarding the total number of diagnosed smear-positive TB patients who started on treatment in 2014 across the city’s 25 TB Units, which are government treatment centers for TB patients. We tabulated these figures to estimate the number and proportion of diagnosed smear-positive TB patients who started on treatment at each TB Unit within the city in 2014.

### Ethical approvals

This analysis is part of a larger study on pretreatment loss to follow-up, which received human subjects approval from the National Institute for Research in TB (NIRT) Institutional Ethics Committee (FWA00005104) on December 29, 2014 and the Brigham and Women’s Hospital (Partners) Institutional Review Board (FWA00000484) on January 13, 2015.

## Results

### Analysis of where TB patients live

#### Findings from 22 DMCs across Chennai

In May 2015, excluding follow-up cases and patients without district or city address listed, a total of 3,543 chest symptomatics were evaluated for suspected pulmonary TB using sputum microscopy and 412 were diagnosed with smear-positive TB at the 22 DMCs in Chennai. The demographic characteristics of these patients are presented in Table 2. About two-thirds of chest symptomatics were men, and the majority of chest symptomatics were evaluated at primary or secondary DMCs. The ages of chest symptomatics evaluated range from 2 to 94 year, with a median age of 43 years and a mean age of 42.8 years. For patients with diagnosed smear-positive TB, the substantial majority (84%) were male, and the majority were evaluated at high-volume DMCs. The ages of diagnosed smear-positive patients ranged from 11 to 77 years, with a median age of 45 years and a mean age of 44.4 years.

**Table 2:** Demographic characteristics of 3,543 patients evaluated for suspected tuberculosis (TB) at 22 designated microscopy centers (DMCs) in Chennai, India.

	Chest symptomatics screened at 22 DMCs (n = 3,543)	Smear-positive TB patients diagnosed at 22 DMCs (n = 412)
	*N (%)*	*N (%)*
**Gender**		
Male	2,349 (66.3)	344 (83.5)
Female	1,194 (33.7)	68 (16.5)
**Age**		
<36	1,214 (34.3)	97 (23.5)
36–50	1,093 (30.8)	182 (44.2)
51+	1,205 (34.0)	132 (32.0)
Not reported	31 (0.9)	1 (0.2)
**Site of initial microscopy test**		
Moderate- or low-volume DMC	1,920 (54.2)	142 (34.5)
High-volume DMC	1,623 (45.8)	270 (65.5)
**Prior TB treatment history**		
New (no prior treatment)	3,269 (92.3)	347 (84.2)
Prior TB treatment	180 (5.1)	61 (14.8)
Not reported	94 (2.7)	4 (1.0)

Of these 3,543 chest symptomatics, 565 (15.9%) had addresses listed outside of Chennai (Table 3). Of the 412 patients at the 22 DMCs diagnosed with smear-positive TB, 71 (17.2%) had addresses outside of Chennai, which is not significantly different from the proportion of chest symptomatics with out-of-city addresses (p = 0.463). A lower proportion of smear-positive patients with a prior TB history had addresses outside of Chennai (8.2%) when compared to the overall population of diagnosed smear-positive patients, though this did not meet statistical significance (p = 0.074).

**Table 3:** Address locations of chest symptomatics and diagnosed smear-positive tuberculosis patients at designated microscopy centers (DMCs) in Chennai, May 2015.

	Chest symptomatics screened at 22 DMCs (n = 3,543)	Smear-positive TB patients diagnosed at 22 DMCs (n = 412)	Smear-positive TB patients with a prior TB history at 22 DMCs (n = 61)	Chest symptomatics screened at high-volume DMCs (n = 1,920)^d^	Smear-positive TB patients diagnosed at high-volume DMCs (n = 270)^d^
	*N (%)*	*N (%)*	*N (%)*	*N (%)*	*N (%)*
**Address within Chennai**	**2,978 (84.1)**^a^	**341 (82.8)**^a^	**56 (91.8)**^a^	**1500 (78.1)**^a^	**216 (80.0)**^a^
600012 (Perambur)	266 (8.9)^c^	34 (10.0)^c^	8 (14.3)^c^	99 (6.6)^c^	20 (9.3)^c^
600021 (Washermanpet)	172 (5.8)^c^	20 (5.9)^c^	7 (12.5)^c^	111(7.4)^c^	16 (7.4)^c^
600019 (Tiruvottiyur)	123 (4.1)^c^	14 (4.1)^c^	1 (1.8)^c^	30 (2.0)^c^	3 (1.4)^c^
600081 (Tondiarpet)	105 (3.5)^c^	7 (2.1)^c^	4 (7.1)^c^	43 (2.9)^c^	5 (2.3)^c^
600039 (Vyasarpadi)	87 (2.9)^c^	13 (3.8)^c^	4 (7.1)^c^	69 (4.6)^c^	11 (5.1)^c^
600004 (Mylapore)	68 (2.3)^c^	12 (3.5)^c^	1 (1.8)^c^	15 (1.0)^c^	6 (2.8)^c^
600031 (Chetpet)	57 (1.9)^c^	5 (1.5)^c^	1 (1.8)^c^	49 (3.3)^c^	4 (1.9)^c^
600002 (Chidatripet)	53 (1.8)^c^	8 (2.3)^c^	1 (1.8)^c^	44 (2.9)^c^	5 (2.3)^c^
600001 (Ft. St. George)	51 (1.7)^c^	6 (1.8)^c^	2 (3.6)^c^	44 (2.9)^c^	5 (2.3)^c^
All other Chennai pincodes	1,392 (46.7)^c^	161 (47.2)^c^	24 (42.9)^c^	733 (48.9)^c^	110 (50.9)^c^
No pincode reported	604 (20.3)^c^	61 (1.8)^c^	3 (5.4)^c^	263 (17.5)^c^	31 (14.3)^c^
**All addresses in districts outside of Chennai**	**565 (15.9)**^a^	**71 (17.2)**^a^	**5 (8.2)**^a^	**420 (21.9)**^a^	**54 (20.0)**^a^
Tiruvallur district	203 (35.9)^b^	31 (43.7)^b^	4 (80.0)^b^	154 (36.7)^b^	25 (46.2)^b^
Vellore district	76 (13.5)^b^	8 (11.3)^b^	0 (0)^b^	52 (12.4)^b^	6 (11.1)^b^
Tiruvannamalai district	58 (10.3)^b^	5 (7.0)^b^	0 (0)^b^	31 (7.4)^b^	7 (13.0)^b^
Kancheepuram district	56 (9.9)^b^	8 (11.3)^b^	1 (20.0)^b^	43 (10.2)^b^	4 (7.4)^b^
Villupuram district	56 (9.9)^b^	6 (8.5)^b^	0 (0)^b^	48 (11.4)^b^	5 (9.3)^b^
Other Tamil Nadu districts	92 (16.3)^b^	11 (15.5)^b^	0 (0)^b^	79 (18.8)^b^	7 (13.0)^b^
Outside of Tamil Nadu	24 (4.2)^b^	2 (2.8)^b^	0 (0)^b^	13 (3.1)^b^	0 (0)^b^

^a^ Percentage is based on the overall sample for each category.

^b^ Percentage is based on the sample of patients with addresses from outside of Chennai for each category.

^c^ Percentage is based on the sample of patients with addresses within Chennai for each category.

^d^ Columns 5 and 6 are not mutually exclusive from Columns 2, 3, and 4; that is, the four high-volume DMCs are a subset of the 22 DMCs.

For both chest symptomatics and diagnosed smear-positive patients from outside of Chennai, the majority came from a few rural districts and towns relatively close to the city—most prominently Tiruvallur district (about 45 km from Chennai) but also Vellore (136 km away), Kancheepuram (70 km away), Tiruvannamallai (180 km away), and Villupuram (160 km away) (Table 3). Only 24 chest symptomatics (4% of those with outside addresses) came from outside of Tamil Nadu state, of whom the majority came from the nearby states of Andhra Pradesh (especially Nellore district, 170 km away), Kerala, Pondicherry, and Karnataka. A few were from states that are substantially farther away, such as Maharashtra, West Bengal, and Sikkim.

**Fig 1:**
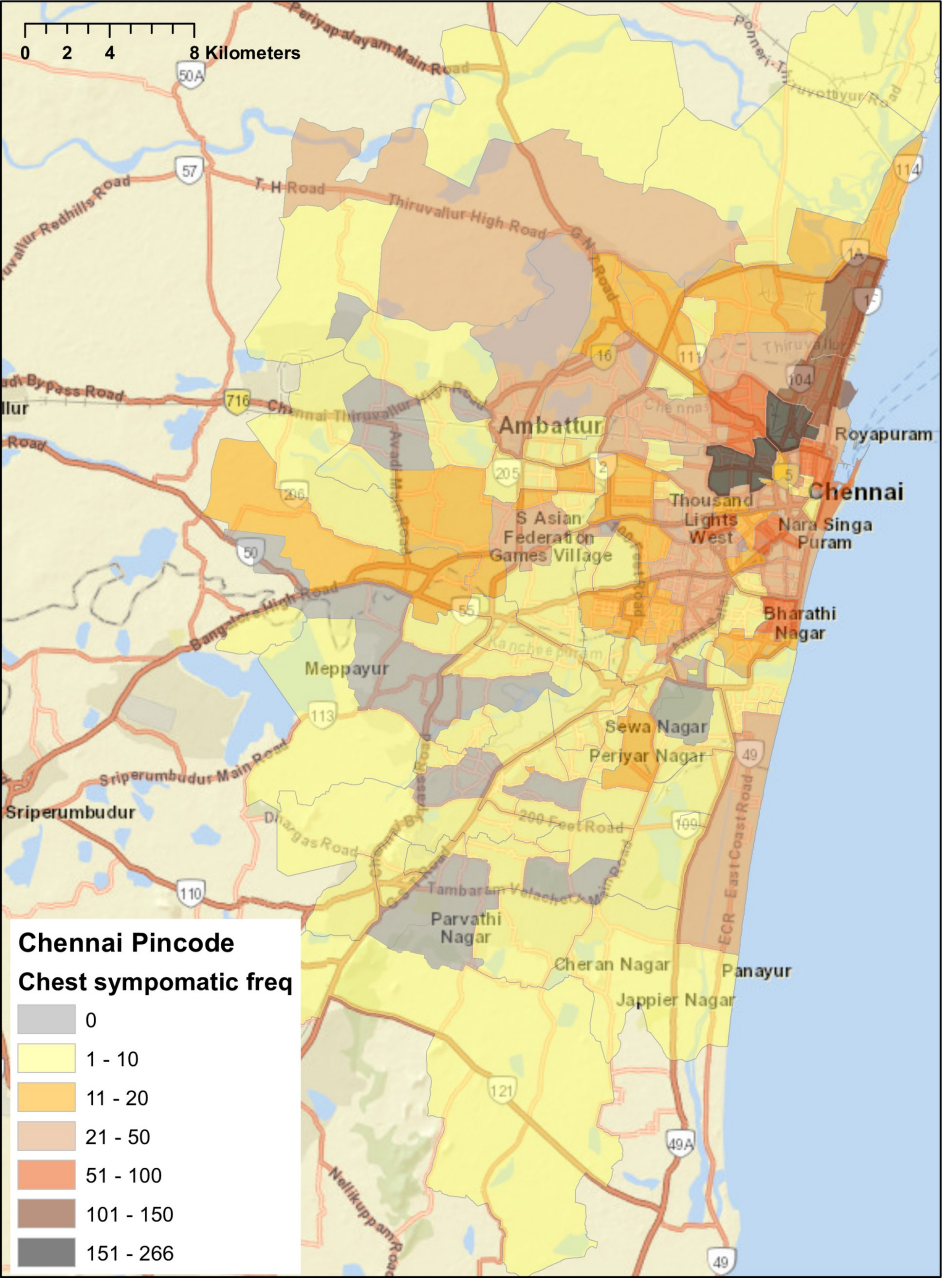
Distribution of home addresses by pincode for chest symptomatics screened for tuberculosis at government designated microscopy centers in Chennai, May 2015. World Street Map base map generated from ArcGIS. Copyright © ArcGIS. All rights reserved.

**Fig 2:**
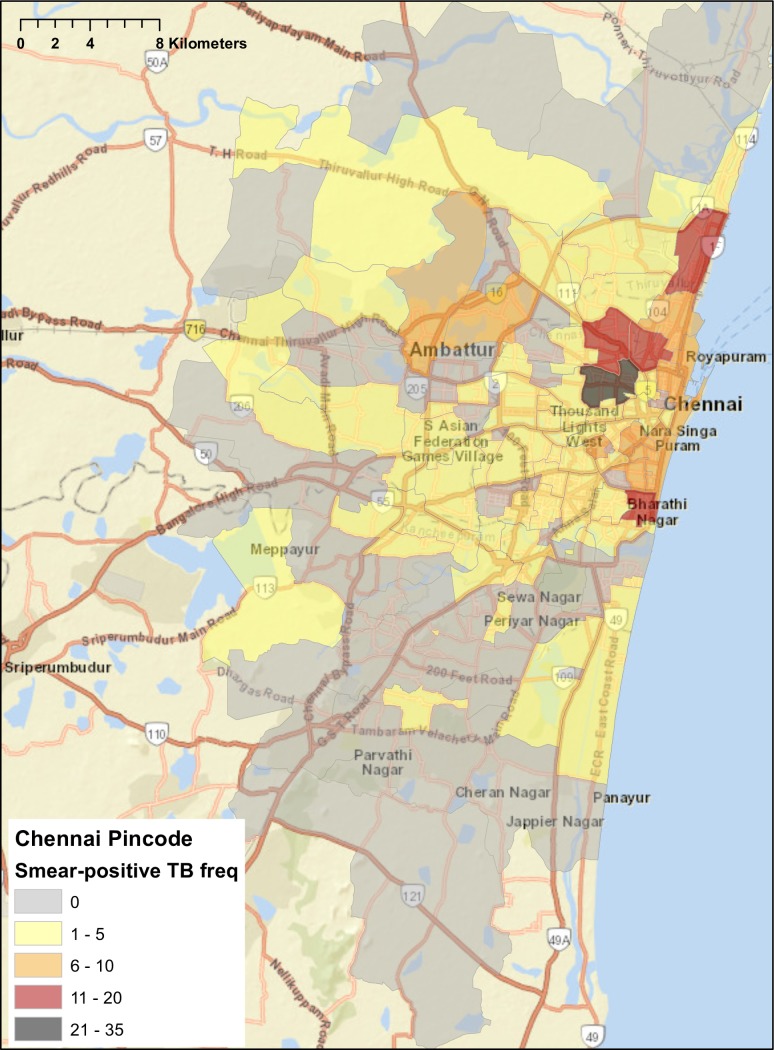
Distribution of home addresses by pincode for smear-positive tuberculosis patients diagnosed at government designated microscopy centers in Chennai, May 2015. World Street Map base map generated from ArcGIS. Copyright © ArcGIS. All rights reserved.

For chest symptomatics and diagnosed smear-positive patients with addresses within Chennai, about 15%—17% of patients did not have a pincode reported (Table 3). The remaining had addresses dispersed across 118 of the city’s 298 pincodes. However, the pincodes with the largest number of both chest symptomatics and diagnosed smear-positive patients clustered within a few areas immediately adjacent to each other in north Chennai, including Perambur, Washermanpet, Tiruvottiyur, Tondiarpet, Vyasarpadi, and Fort St. George (Table 3, Figs 1 and 2).

#### Findings from the four high-volume DMCs in Chennai

Out of 1,920 chest symptomatics screened for TB at the four high-volume DMCs, 420 (21.9%) had out-of-city addresses (Table 3), which is significantly higher than the 145 out of 1,623 (8.9%) chest symptomatics screened for TB at the remaining 18 medium- or low-volume DMCs with out-of-city addresses (p<0.0001). Out of 270 smear-positive patients diagnosed at the high-volume centers, 54 (20.0%) had addresses located outside of Chennai (Table 3), which is significantly higher than the 17 (12.0%) of 142 smear-positive patients diagnosed at the remaining 18 medium- or low-volume DMCs with outside addresses (p = 0.041).

We also disaggregated data on the address locations of chest symptomatics and diagnosed smear-positive patients by each of the four high-volume DMCs (Table 4). Chennai General Hospital (i.e., Madras Medical College) had a considerably higher proportion of both of chest symptomatics (43.2%) and diagnosed smear-positive patients (52.5%) with out-of-city addresses compared to the other high-volume DMCs and the 18 medium- or low-volume DMCs (all p<0.001).

**Table 4:** Patients with addresses located outside of Chennai at the four high-volume designated microscopy centers (DMCs).

High-volume DMC	Chest symptomatics with out-of-city addresses	Smear-positive TB patients with out-of-city addresses
	*N (%)*	*N (%)*
Govt. Thiruvatteeswarar Hosp. of Thoracic Medicine (“Otteri Hospital”)	55/407 (13.5)^a^	16/108 (14.8)^b^
Institute of Thoracic Medicine	46/547 (8.4)^a^	4/65 (6.2)^b^
Chennai General Hospital	265/614 (43.2)^a^	31/59 (52.5)^b^
Government Stanley Hospital	54/352 (15.3)^a^	4/38 (10.5)^b^

^a^ Percent out of all chest symptomatics at the specific high-volume DMC

^b^ Percent out of all diagnosed smear-positive patients at the specific high-volume DMC

Notably, of all 565 chest symptomatics with addresses located outside of Chennai 420 (74.3%) were evaluated at the four high-volume DMCs. Chennai General Hospital alone evaluated 265 (46.9%) of all chest symptomatics with out-of-city addresses. Of the 71 diagnosed smear-positive patients with an address outside of Chennai, 54 (76.1%) were diagnosed at the high-volume DMCs and Chennai General Hospital alone diagnosed 31 (43.7%) of all smear-positive patients with outside addresses.

#### Analysis of where TB patients are evaluated and diagnosed

In 2014, out of all 54 city DMCs, the four high-volume DMCs evaluated 27,779 (40.1%) of the total 69,200 chest symptomatics who underwent TB screening, and these high-volume DMCs diagnosed 3,498 (57%) of 6,135 total smear-positive patients. The next 18 medium- or low-volume DMCs screened 27,274 (39.4%) of the total 69,200 chest symptomatics and diagnosed 2,022 (33.0%) of 6,135 total smear-positive patients (Table 5). The remaining 32 lowest volume DMCs screened 14,147 (20.4%) of 69,200 chest symptomatics and diagnosed only 615 (10.0%) of 6,135 total smear-positive patients.

**Table 5:** Distribution of chest symptomatics, newly diagnosed smear-positive TB patients, and smear-positive case detection rates at 22 designated microscopy centers in Chennai, 2014.

Name of facility	TB suspects evaluated (n = 69,200)	Smear-positive TB cases diagnosed (n = 6,135)	Percent of TB suspects with positive smears (case detection rate)
	*N (%)*^a^	*N (%)*^b^	*%*
Otteri TB Hospital	6,381 (9.2)	1,317 (21.5)	20.6
Institute of Thoracic Med.	9,478 (13.7)	798 (13.0)	8.4
Madras Medical College	7,132 (10.3)	779 (12.7)	10.9
Stanley Hospital	4,788 (6.9)	604 (9.8)	12.6
Royapettah Hospital	3,133 (4.5)	306 (5.0)	9.8
GPH, KK Nagar	1,778 (2.6)	231 (3.8)	13.0
Sri Ramachandra Medical College	1,892 (2.7)	196 (3.2)	10.4
Pulianthope TB Clinic	2,274 (3.3)	187 (3.0)	9.2
Kilpauk Medical College	4,727 (6.8)	178 (2.9)	3.8
Communicable Disease Hospital	1,618 (2.3)	119 (1.9)	7.4
Thiruvanmiyur UPHC	990 (1.4)	103 (1.7)	10.4
Basin Bridge UPHC	1,131 (1.6)	97 (1.6)	8.6
GPH, Tondiarpet	1,185 (1.7)	92 (1.5)	7.8
GPH, Anna Nagar	1,356 (2.0)	70 (1.1)	5.2
Thiruvetriyur	836 (1.2)	69 (1.1)	8.3
Saidapet General Hospital	1,099 (1.6)	67 (1.1)	6.1
Kodambakkam UPHC	921 (1.3)	62 (1.0)	6.7
Thanthai Periyar UPHC	842 (1.2)	59 (1.0)	7.0
ESI Hospital, Ayanavaram	1,511 (2.2)	58 (0.9)	3.8
Nungambakkam UPHC	867 (1.3)	47 (0.8)	5.4
GPH, Periyar Nagar	676 (1.0	45 (0.7)	6.7
Mylapore UPHC	438 (0.6)	36 (0.6)	8.2

TB = tuberculosis; GPH = government public hospital; UPHC = universal primary health center.

^a^ This percentage represents the number of people with suspected TB evaluated at each facility divided by the total number of 69,200 people with suspected TB evaluated in Chennai in 2014; data for the 32 centers with the lowest patient volume are not shown.

^b^ This percentage represents the number of smear-positive TB patients diagnosed at each facility divided by the total number of 6,135 smear-positive TB patients diagnosed in Chennai in 2014; data for the 32 centers with the lowest patient volume are not shown.

The smear-positive case detection rate—the proportion of chest symptomatics screened who had a positive sputum smear—varied substantially across DMCs, from as high as 20.6% at Government Thiruvatteeswarar (Otteri) Hospital of Thoracic Medicine to as low as 0.2% at some primary health centers and the Institute of Child Health. The four high-volume DMCs had a pooled smear-positive case detection rate of 12.6%. The subsequent 18 DMCs with medium- to low-volume had a pooled smear-positive case detection rate of 7.4%, which is significantly lower than the case detection rate at the high-volume DMCs (p<0.0001). The 32 lowest-volume DMCs had a pooled smear-positive case detection rate of 4.3%, which is significantly lower than the case detection rate in both the four high-volume DMCs and the 18 medium- or low-volume DMCs (both p<0.0001).

### Analysis of where TB patients start treatment

In 2014, total of 3,276 smear-positive patients (2,778 new and 498 retreatment patients) registered for TB treatment in Chennai in the 25 TB Units (Table 6). The distribution of the proportion of patients treated at each TB Unit varied from 1.8% at Lalithapuram to 7.9% at East Cemetery Road. In general, the distribution of patient volume was relatively similar across TB Units, with most treating between 2%—6% of the city’s TB patients.

**Table 6:** Distribution of smear-positive tuberculosis (TB) patients being treated at different TB Units in Chennai, 2014.

TB Unit	Number of smear-positive patients treated *N (%)*^a^
East Cemetery Road	260 (7.9)
Villivakkam	239 (7.3)
Tondaiyarpet	216 (6.6)
Basin Bridge	215 (6.6)
Elango Nagar	192 (5.9)
Velachery	190 (5.8)
Kodugaiyur	182 (5.6)
Ice House	142 (4.3)
Thanthai Periyar	140 (4.3)
Pulianthope	129 (3.9)
MMDA	127 (3.9)
Cindadripet	123 (3.8)
Kodambakkam	119 (3.6)
Thiruvanmiyur	119 (3.6)
Nungambakkam	114 (3.5)
Kotturpuram	109 (3.3)
Thiru-vi-ka Nagar	103 (3.1)
Mylapore	91 (2.8)
Aminjikarai	90 (2.7)
Saidapet West	90 (2.7)
Virugambakkam	81 (2.5)
Teynampet	78 (2.4)
Kolathur	67 (2.0)
Lalithapuram	60 (1.8)

^a^Percent out of the overall 3,276 smear-positive TB patients started on treatment in Chennai in 2014.

## Discussion

Our findings suggest that the TB “urban registration gap” in Chennai may be partly explained by the temporary movement of patients from rural areas and towns into the city for diagnostic evaluation, especially at high-volume DMCs, which represent major tertiary hospitals or TB specialty facilities in the city. About one-sixth of all chest symptomatics and smear-positive patients reported home addresses located outside of Chennai city, mostly in nearby districts in Tamil Nadu state. In the RNTCP, regardless of where TB patients are diagnosed, they are referred to receive treatment at the DOT center closest to their homes, to facilitate the monitoring strategy of thrice-weekly facility-based directly observed therapy. As such, most patients with addresses outside of Chennai were likely referred back to their rural districts to start treatment and would not show up in Chennai’s TB treatment statistics. Indeed, 2013 RNTCP statistics suggest that in Tiruvallur—the district where the most out-of-city patients came from—only 1,815 patients were diagnosed with smear-positive TB at DMCs within the district, while 2,333 smear-positive TB patients were registered for TB treatment within the district [3]. This suggests that more than one-fifth of smear-positive TB patients on treatment in Tiruvallur may have been diagnosed outside of the district, likely in Chennai.

However, the 17% of diagnosed smear-positive patients with out-of-city addresses only explains about one-third of Chennai’s urban registration gap. It is possible that we underestimate the number of patients who live outside of Chennai, because some patients who migrate to Chennai for diagnostic evaluation may stay with relatives or friends in the city and report local addresses within the city when submitting sputum samples. However, it is unlikely that the phenomenon of out-of-city patients reporting local addresses within the city would explain the entire urban registration gap.

As such, our findings have potentially important implications for the problem of pretreatment loss to follow-up (“initial default”) of newly diagnosed TB patients. First, since out-of-city patients only explain a small proportion of the urban registration gap, it is possible that the remaining gap is explained by pretreatment loss to follow-up of newly diagnosed TB patients who live within Chennai. Second, we anticipate that out-of-city TB patients might be at increased risk for pretreatment loss to follow-up, due to the potential challenges involved in traveling long distances to the city to get diagnosed and then having to return to rural areas to start treatment. We are currently conducting a cohort study that involves intensive tracking of newly diagnosed smear-positive TB patients to evaluate the problem of pretreatment loss to follow-up in Chennai in greater detail. We anticipate that the forthcoming results of that study, along with the findings of high patient mobility in this current study, will provide a more complete explanation for the urban registration gap.

Pretreatment loss to follow-up is a major concern for TB control, as these patients are at high risk for death and may transmit TB to others [8, 9]. Recent meta-analyses of previously published local studies estimated overall pretreatment loss to follow-up rates of 16% in India [2] and 18% in sub-Saharan African countries [10]. Factors contributing to pretreatment loss to follow-up in Indian studies included alcohol use disorder [11], TB-related stigma [11], employment-related barriers [12, 13], and dissatisfaction with government health services [13, 14]. In addition, multiple studies found rural-to-urban migration of patients and distance of patients’ homes from DMCs to be major contributing factors to pretreatment loss to follow-up [12, 14, 15].

In the current study, about three-fourths of patients from outside of Chennai sought care at high-volume DMCs in the city, likely because these hospitals are well known. For example, Chennai General Hospital has one of the state’s best-known medical colleges. It is also located across the street from the city’s largest railway station, making it easily accessible to out-of-city patients. About half of all chest symptomatics and smear-positive patients at Chennai General Hospital had out-of-city addresses. A recent study at a TB specialty treatment center near Chennai (Government Hospital of Thoracic Medicine in Tambaram) found that many patients bypass their local TB services to come to these facilities because of name recognition, perceptions that they will receive higher quality care, and referral by other TB patients [16]. Supporting the idea that many patients are bypassing local services is the fact that TB infrastructural coverage in many rural districts is fairly robust. For example, Tiruvallur district (which contributed the most out-of-city patients) has more than 30 DMCs.

While Chennai’s RNTCP has regular meetings to facilitate communication between staff at TB facilities within the city to ensure appropriate referral and tracking of patients, these same routine communication mechanisms are not available between DMCs in Chennai and TB Units in other districts. Major tertiary hospitals and TB specialty facilities in urban centers like Chennai need to develop robust services for linking rural TB patients to care and need to develop processes for tracking these patients to ensure they reach rural treatment sites.

Novel strategies to improve patient tracking might leverage India’s Unique Identification (UID or Aadhar) system, which includes biometric information (i.e., fingerprints and iris scans) and is estimated to have enrolled about 85% of India’s population. A UID-based biometric-driven patient registration system at all TB facilities at a state or national level would avoid duplication of patient information and help to track patients who are referred to sites both within and outside of the city to ensure they are linked to care. Such a system would help to accurately estimate true patient losses across multiple steps of the TB cascade of care [17]. Also, the use of patient navigators and case managers has been helpful in maintaining continuity of care for people living with HIV and might be worth evaluating for TB patients in the Indian context [18].

Mapping the addresses of patients located within Chennai suggests that a considerable proportion of TB patients are coming from a few pincodes in North Chennai. In theory, this finding could be due to detection bias, since North and Central Chennai have more TB diagnostic services and public hospitals than South Chennai. Additionally, maps of TB patients were not able take into consideration the population size within each pincode, which would allow for an examination of TB rates given different population distributions across the city.

However, these North Chennai pincodes have a high population density and considerable overcrowding, and they are among the poorest areas of the city. In addition, our findings regarding the higher burden of TB in North Chennai is consistent with results of a recent city-wide TB prevalence survey conducted from 2010–2012 [19]. Our findings support the results of the prevalence survey to suggest that the densely populated areas of Perambur, Washermanpet, Tiruvottiyur, Tondiarpet, Vyasarpadi, and Fort St. George have a high burden of TB and should be geographical focus areas for TB public health interventions, such as population- and health facility-based active case finding and community education so that people with chest symptoms present earlier to DMCs.

We find that diagnosis of TB patients in Chennai is highly centralized, with just four high-volume DMCs diagnosing 57% of all smear-positive patients and the 22 highest-volume DMCs diagnosing 90% of all smear-positive patients. In contrast, there was a relatively equitable distribution of TB patients starting treatment across the city’s 25 TB Units. On the one hand, this finding highlights a need for the RNTCP to improve the general public’s awareness of local TB diagnostic services, especially for rural patients migrating hundreds of kilometers in some cases to seek diagnostic evaluation in Chennai’s tertiary hospitals or TB specialty facilities, when their local facilities should be able to readily diagnose TB.

On the other hand, the highly centralized flow of patients offers unique opportunities for strengthening TB care delivery, because strategic interventions at these critical high-volume DMCs could rapidly impact a large proportion of the city’s TB patients. For example, at the time of our study, there was only one light microscopist evaluating sputum samples at each of the four high-volume DMCs, despite the fact that these DMCs diagnose nearly six times the number of smear-positive patients as the 32 lowest-volume DMCs. Increasing the number of light microscopists at each of these DMCs could facilitate same-day TB diagnosis and treatment initiation [20], which may improve linkage to care [21].

In addition, strategic placement of new diagnostic tests such as CB-NAATs for upfront testing of chest symptomatics at these high-volume sites could rapidly increased diagnosis of smear-negative and drug-resistant TB patients. A recent systematic review suggests that there is considerable underdiagnosis of smear-negative TB in the RNTCP, as the majority of patients do not complete the multi-step workup for empiric TB diagnosis [2]. Currently, drug-resistance testing in Chennai is mostly performed using line-probe assay only on retreatment TB patients or HIV patients with positive smears, which reduces the proportion of drug-resistant patients who can be diagnosed and increases their time to diagnosis. A recent study of the implementation of CB-NAATs for upfront testing at select RNTCP sites showed a dramatic increase in diagnosis of both smear-negative and drug-resistant TB patients [22]. The high smear-positive case detection rate at Chennai’s high-volume DMCs—especially Otteri Hospital, where 21% of chest symptomatics were smear-positive—suggests that use of CB-NAATs upfront selectively at these sites may have greater value and cost-effectiveness compared to its use at sites with lower case detection rates.

The smear-positive case detection rate may be higher at DMCs in tertiary hospitals or TB specialty facilities (compared to rates in DMCs in primary or secondary health centers) because some patients may have been referred to these hospitals by other public or private sector providers due to the severity of their illness. As such, these patients may be more likely to have TB or may have more severe disease due to health system-related delays in diagnosis [23]. Alternatively, differences in case detection rates could partly represent differences in the quality of evaluation by laboratory microscopists, with higher volume centers possibly having more skilled technicians who are better able to detect patients with lower acid-fast bacilli burdens.

Other major cities in India have a considerable urban TB registration gap (as described in the Introduction), so future research should investigate whether patient mobility is a common phenomenon in other cities. The last Indian study we could find that addressed this question, conducted in Bengaluru in 1963, found that about 17% of diagnosed TB patients resided outside of the city, which is similar to the current study [24].

Future research should investigate whether centralization of diagnostic volume at major tertiary hospitals or TB specialty facilities is also common in other cities. Mumbai is one of the few cities to disaggregate its TB reporting to the RNTCP into 22 different city areas, rather than reporting statistics for the city as a whole [3]. These data show that just four of these city areas—which contain the largest public tertiary hospitals in the city—diagnose about 43% of the city’s smear-positive patients, which may also suggest considerable centralization of diagnostic volume at a few tertiary hospitals (Table 7) [3]. If patient mobility and centralized diagnosis are common issues in other cities, the RNTCP could consider larger scale initiatives focused on improving diagnosis and linkage to care for rural patients visiting tertiary hospitals and TB specialty facilities across major cities in India. Future research should also seek to elucidate the healthcare-seeking behavior of these patients from rural areas, especially by conducting qualitative interviews, to understand why rural patients may be bypassing local TB services and traveling long distances to seek care in the city.

**Table 7:** Four areas (out of 22 total city areas) with the largest numbers of smear-positive tuberculosis diagnoses and nearby tertiary hospitals and TB specialty facilities in Mumbai, 2013.

Area of Mumbai	Nearby tertiary hospital or TB specialty facility	Smear-positive TB patients diagnosed in Mumbai in 2013 *N (%)*^a^
Parel	King Edward Memorial Hospital	2,844 (19.9)
Dadar	Sewri TB Hospital	1,217 (8.5)
Byculla	JJ Hospital	1,031 (7.2)
Sion	Lokmanya Tilak (“Sion”) Hospital	1,048 (7.3)

^a^Percentage is out of a total of 14,269 smear-positive TB patients diagnosed in Mumbai in 2013 [3].

Our study has a few important limitations. First, to make this study feasible, we were not able to audit the May 2015 records for 32 of the 54 DMCs in Chennai; however, these 32 DMCs diagnosed only about 10% of the city’s smear-positive patients given their low patient volume and case detection rates. Second, about 4% of all DMC register entries for patients did not list an address, so we could not include them in this analysis. It is possible that both of the above limitations might bias us towards overestimating the proportion of out-of-city chest symptomatics and smear-positive patients. However, we more likely underestimated the proportion of out-of-city patients, because some patients from other districts may list a local relative or friend’s address within Chennai, even if they are only in the city temporarily.

## Conclusions

In this study, we found that about one-sixth of all TB patients diagnosed at DMCs in Chennai have home addresses located outside of the city, and this finding is inadequate to explain Chennai’s entire urban registration gap, suggesting that there may be a considerable pretreatment loss to follow-up rate in the city. We also found that diagnosis of TB patients is highly centralized, with four high-volume DMCs diagnosing the majority of smear-positive TB patients in the city. The RNTCP should ensure strong coordination between high-volume DMCs in Chennai and the rural TB Units where these patients are likely to take treatment. Novel strategies such as a Unique Identification-based, biometric-driven patient registration system might help with this goal of improving patient tracking and linkage to care.

In addition, introduction of new diagnostic tests such as CB-NAATs at major tertiary hospitals and TB specialty facilities for upfront screening of patients could have an impact on a large proportion of the city’s TB patients. Re-distribution of RNTCP personnel in different DMCs depending on the burden of TB patients diagnosed each facility may result in more efficient utilization of resources and better quality of care.

Future studies should aim to understand why rural patients are coming to Chennai for diagnostic evaluation rather than going to TB facilities in their local districts, potentially through qualitative interviews. In addition, a needs assessment for these out-of-city patients may help to understand how TB care can be improved for this population. Finally, the urban registration gap is a phenomenon in several cities and future studies of patient addresses and pretreatment loss to follow-up in other cities may help improve urban TB care in India.

## Supporting information

S1 Appendix Dataset for where Chennai’s TB patients live and get diagnosed. (XLSX)

S2 Appendix Data dictionary accompanying the dataset for where Chennai’s TB patients live and get diagnosed. (PDF)

S3 Appendix OpenStreetMap license information. (PDF)
