# Correction: Tuberculosis patients in an Indian mega-city: Where do they live and where are they diagnosed?

**DOI:** 10.1371/journal.pone.0224808

**Published:** 2019-10-30

**Authors:** Ramnath Subbaraman, Beena E. Thomas, Senthil Sellappan, Chandra Suresh, Lavanya Jayabal, Savari Lincy, Agnes L. Raja, Allison McFall, Sunil Suhas Solomon, Kenneth H. Mayer, Soumya Swaminathan

## Notice of Republication

Incorrect versions of Fig 1 and Fig 2 were published in error. This article was republished on October 23, 2019 to correct for these errors. Please download this article again to view the correct version.
