# Supplementary material for: Tuberculosis patients in an Indian mega-city: Where do they live and where are they diagnosed?
Source: PLoS One. 2017 Aug 15;12(8):e0183240. doi: 10.1371/journal.pone.0183240 (PMC5557603; doi:10.1371/journal.pone.0183240)
Supplement: S2 Appendix — (PDF) [file pone.0183240.s002.pdf]

Data dictionary for “Tuberculosis Patients in an Indian Mega-city: Where Do They Live and Where Are They Diagnosed?”

1. record_id: A unique number is listed for each patient.

2. dmc_site:

1	Otteri TB Hospital
2	Institute of Thoracic Med.
3	Madras Medical College
4	Govt. Stanley Hospital
5	Govt. Royapettah Hospital
6	GPH KK Nagar
7	Sri Ramachandra Medical College
8	Pulianthope TB Clinic
9	Kilpauk Medical College
10	Communicable Disease Hospital
11	Thiruvanmiyur UPHC
12	Basin Bridge UPHC
13	GPH Tondiarpet
14	GPH Anna Nagar
15	Thiruvetriyur
16	Saidapet General Hospital
17	Kodambakkam UPHC
18	Thanthai Periyar UPHC
19	ESI Hospital Ayanavaram
20	Nungambakkam UPHC
21	GPH Periyar Nagar
22	Mylapore UPHC

3. age: age in years as listed.

4. gender:

1	Male
2	Female
3	Transgender

5. district_in_tn: refers to the "district in Tamil Nadu" in which the patient's address is located

1	Chennai
2	Ariyalur
3	Coimbatore
4	Cuddalore
5	Dharmapuri
6	Dindigul
7	Erode
8	Kancheepuram
9	Kanyakumari
10	Karur
11	Krishnagiri
12	Madurai
13	Nagapattinam
14	Namakkal
15	Perambalur
16	Pudukkottai
17	Ramanathapuram
18	Salem
19	Sivaganga
20	Thanjavur
21	The Nilgiris
22	Theni
23	Thiruvallur
24	Thiruvarur
25	Thoothukkudi
26	Tiruchirapalli
27	Tirunelveli
28	Tiruppur
29	Tiruvannamalai
30	Vellore
31	Villupuram
32	Virudhunagar
33	Unknown district
34	Outside of Tamil Nadu

6. outside_tn_location: for patients with addresses outside of Tamil Nadu state, this is a free text entry providing information on the location (usually the state and/or city) where the patient's address is located

7. pin_code: refers to the "postal index number" listed with the patient's address, which provides a more specific area for each patient's address within each district or city

8. reason_for_exam: provides information on the reasons for screening each patient, including information on whether the sputum is for follow-up evaluation (for patients already on treatment) or whether the patient is a "new" patient who has never had TB before or whether the patient has a history of prior TB therapy. Note that all "follow-up" patients have already been excluded from this dataset.

1	New
2	Prior treatment
3	Follow-up
4	Not listed

9. sputum_a_result: refers to whether the first "spot" sputum sample submitted by the patient was positive, and, if so, the "grade" of sputum positivity

0	negative
1	1+
2	2+
3	3+
4	Scant
XXX	Not listed

10. sputum_b_result: refers to whether the second "morning" sputum sample submitted by the patient was positive, and, if so, the "grade" of sputum positivity

0	negative
1	1+
2	2+
3	3+
4	Scant
XXX	Not listed

11. Overall status—sputum positive or negative: refers to the overall status of the patient. If the patient had at least one positive sputum smear (1+, 2+, 3+, or scant), she or he is considered to have a diagnosis of smear-positive TB. If both smears are negative for the patient or not listed, the patient requires further workup for possible smear-negative TB.

1	At least one positive sputum sample (presumed smear-positive TB)
2	Both smears are either negative or not listed
