# Supplementary material for: Tuberculosis patients in an Indian mega-city: Where do they live and where are they diagnosed?
Source: PLoS One. 2017 Aug 15;12(8):e0183240. doi: 10.1371/journal.pone.0183240 (PMC5557603; doi:10.1371/journal.pone.0183240)
Supplement: S3 Appendix — (PDF) [file pone.0183240.s003.pdf]

Copyright and License

OpenStreetMap® is *open data*, licensed under the Open Data Commons Open Database License (ODbL) by the OpenStreetMap Foundation (OSMF).

You are free to copy, distribute, transmit and adapt our data, as long as you credit OpenStreetMap and its contributors. If you alter or build upon our data, you may distribute the result only under the same licence. The full legal code explains your rights and responsibilities.

The cartography in our map tiles, and our documentation, are licensed under the Creative Commons Attribution-ShareAlike 2.0 license (CC BY-SA).

How to credit OpenStreetMap

We require that you use the credit "© OpenStreetMap contributors".

You must also make it clear that the data is available under the Open Database License, and if using our map tiles, that the cartography is licensed as CC BY-SA. You may do this by linking to this copyright page. Alternatively, and as a requirement if you are distributing OSM in a data form, you can name and link directly to the license(s). In media where links are not possible (e.g. printed works), we suggest you direct your readers to openstreetmap.org (perhaps by expanding 'OpenStreetMap' to this full address), to opendatacommons.org, and if relevant, to creativecommons.org.

For a browsable electronic map, the credit should appear in the corner of the map. For example:

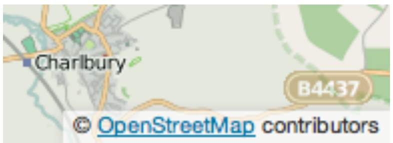

Finding out more

Read more about using our data, and how to credit us, at the OSMF Licence page and the community Legal FAQ.

Although OpenStreetMap is open data, we cannot provide a free-of-charge map API for third-parties. See our API Usage Policy, Tile Usage Policy and Nominatim Usage Policy.

Our contributors

Our contributors are thousands of individuals. We also include openly-licensed data from national mapping agencies and other sources, among them:

- **Austria**: Contains data from Stadt Wien (under CC BY), Land Vorarlberg and Land Tirol (under CC BY AT with amendments).
- **Canada**: Contains data from GeoBase®, GeoGratis (© Department of Natural Resources Canada), CanVec (© Department of Natural Resources Canada), and StatCan (Geography Division, Statistics Canada).
- **Finland**: Contains data from the National Land Survey of Finland's Topographic Database and other datasets, under the NLSFI License.
- **France**: Contains data sourced from Direction Générale des Impôts.
- **Netherlands**: Contains © AND data, 2007 (www.and.com)
- **New Zealand**: Contains data sourced from Land Information New Zealand. Crown Copyright reserved.

- **Slovenia**: Contains data from the Surveying and Mapping Authority and Ministry of Agriculture, Forestry and Food (public information of Slovenia).
- **South Africa**: Contains data sourced from Chief Directorate: National Geo-Spatial Information, State copyright reserved.
- **United Kingdom**: Contains Ordnance Survey data © Crown copyright and database right 2010-12.

For further details of these, and other sources that have been used to help improve OpenStreetMap, please see the Contributors page on the OpenStreetMap Wiki.

Inclusion of data in OpenStreetMap does not imply that the original data provider endorses OpenStreetMap, provides any warranty, or accepts any liability.

Copyright infringement

OSM contributors are reminded never to add data from any copyrighted sources (e.g. Google Maps or printed maps) without explicit permission from the copyright holders.

If you believe that copyrighted material has been inappropriately added to the OpenStreetMap database or this site, please refer to our takedown procedure or file directly at our on-line filing page.

Trademarks

OpenStreetMap, the magnifying glass logo and State of the Map are registered trademarks of the OpenStreetMap Foundation. If you have questions about your use of the marks, please send your questions to the Licence Working Group.

Open Data Commons *Legal tools for Open Data*

Open Database License (ODbL) v1.0

Disclaimer

Open Data Commons is not a law firm and does not provide legal services of any kind.

Open Data Commons has no formal relationship with you. Your receipt of this document does not create any kind of agent-client relationship. Please seek the advice of a suitably qualified legal professional licensed to practice in your jurisdiction before using this document.

No warranties and disclaimer of any damages. This information is provided 'as is', and this site makes no warranties on the information provided. Any damages resulting from its use are disclaimed.

Plain language summary

A plain language summary of the Open Database License is available.

Alternative formats:

Plain Text

ODC Open Database License (ODbL)

Preamble

The Open Database License (ODbL) is a license agreement intended to allow users to freely share, modify, and use this Database while maintaining this same freedom for others. Many databases are covered by copyright, and therefore this document licenses these rights. Some jurisdictions, mainly in the European Union, have specific rights that cover databases, and so the ODbL addresses these rights, too. Finally, the ODbL is also an agreement in contract for users of this Database to act in certain ways in return for accessing this Database.

Databases can contain a wide variety of types of content (images, audiovisual material, and sounds all in the same database, for example), and so the ODbL only governs the rights over the Database, and not the contents of the Database individually. Licensors should use the ODbL together with another license for the contents, if the contents have a single set of rights that uniformly covers all of the contents. If the contents have multiple sets of different rights, Licensors should describe what rights govern what contents together in the individual record or in some other way that clarifies what rights apply.

Sometimes the contents of a database, or the database itself, can be covered by other rights not addressed here (such as private contracts,

trade mark over the name, or privacy rights / data protection rights over information in the contents), and so you are advised that you may have to consult other documents or clear other rights before doing activities not covered by this License.

The Licensor (as defined below)

and

You (as defined below)

agree as follows:

1.0 Definitions of Capitalised Words

"Collective Database" – Means this Database in unmodified form as part of a collection of independent databases in themselves that together are assembled into a collective whole. A work that constitutes a Collective Database will not be considered a Derivative Database.

"Convey" – As a verb, means Using the Database, a Derivative Database, or the Database as part of a Collective Database in any way that enables a Person to make or receive copies of the Database or a Derivative Database. Conveying does not include interaction with a user through a computer network, or creating and Using a Produced Work, where no transfer of a copy of the Database or a Derivative Database occurs.
"Contents" – The contents of this Database, which includes the information, independent works, or other material collected into the Database. For example, the contents of the Database could be factual data or works such as images, audiovisual material, text, or sounds.

"Database" – A collection of material (the Contents) arranged in a systematic or methodical way and individually accessible by electronic or other means offered under the terms of this License.

"Database Directive" – Means Directive 96/9/EC of the European Parliament and of the Council of 11 March 1996 on the legal protection of databases, as amended or succeeded.

"Database Right" – Means rights resulting from the Chapter III ("sui generis") rights in the Database Directive (as amended and as transposed by member states), which includes the Extraction and Re-utilisation of the whole or a Substantial part of the Contents, as well as any similar rights available in the relevant jurisdiction under Section 10.4.

"Derivative Database" – Means a database based upon the Database, and includes any translation, adaptation, arrangement, modification, or any other alteration of the Database or of a Substantial part of the Contents. This includes, but is not limited to, Extracting or Re-utilising the whole or a Substantial part of the Contents in a new Database.

"Extraction" – Means the permanent or temporary transfer of all or a Substantial part of the Contents to another medium by any means or in any form.

"License" – Means this license agreement and is both a license of rights such as copyright and Database Rights and an agreement in contract.

"Licensor" – Means the Person that offers the Database under the terms of this License.

"Person" – Means a natural or legal person or a body of persons corporate or incorporate.

"Produced Work" – a work (such as an image, audiovisual material, text, or sounds) resulting from using the whole or a Substantial part of the Contents (via a search or other query) from this Database, a Derivative Database, or this Database as part of a Collective Database.

"Publicly" – means to Persons other than You or under Your control by either more than 50% ownership or by the power to direct their activities (such as contracting with an independent consultant).

"Re-utilisation" – means any form of making available to the public all or a Substantial part of the Contents by the distribution of copies, by renting, by online or other forms of transmission.

"Substantial" – Means substantial in terms of quantity or quality or a combination of both. The repeated and systematic Extraction or Re-utilisation of insubstantial parts of the Contents may amount to the Extraction or Re-utilisation of a Substantial part of the Contents.

"Use" – As a verb, means doing any act that is restricted by copyright or Database Rights whether in the original medium or any other; and includes without limitation distributing, copying, publicly performing, publicly displaying, and preparing derivative works of the Database, as well as modifying the Database as may be technically necessary to use it in a different mode or format.

"You" – Means a Person exercising rights under this License who has not previously violated the terms of this License with respect to the Database, or who has received express permission from the Licensor to exercise rights under this License despite a previous violation.

Words in the singular include the plural and vice versa.

2.0 What this License covers

2.1. Legal effect of this document. This License is:

a. A license of applicable copyright and neighbouring rights;

b. A license of the Database Right; and

c. An agreement in contract between You and the Licensor.

2.2 Legal rights covered. This License covers the legal rights in the Database, including:

a. Copyright. Any copyright or neighbouring rights in the Database. The copyright licensed includes any individual elements of the Database, but does not cover the copyright over the Contents independent of this Database. See Section 2.4 for details. Copyright law varies between jurisdictions, but is likely to cover: the Database

model or schema, which is the structure, arrangement, and organisation of the Database, and can also include the Database tables and table indexes; the data entry and output sheets; and the Field names of Contents stored in the Database;

b. Database Rights. Database Rights only extend to the Extraction and Re-utilisation of the whole or a Substantial part of the Contents. Database Rights can apply even when there is no copyright over the Database. Database Rights can also apply when the Contents are removed from the Database and are selected and arranged in a way that would not infringe any applicable copyright; and

c. Contract. This is an agreement between You and the Licensor for access to the Database. In return you agree to certain conditions of use on this access as outlined in this License.

2.3 Rights not covered.

a. This License does not apply to computer programs used in the making or operation of the Database;

b. This License does not cover any patents over the Contents or the Database; and

c. This License does not cover any trademarks associated with the Database.

2.4 Relationship to Contents in the Database. The individual items of the Contents contained in this Database may be covered by other rights, including copyright, patent, data protection, privacy, or personality rights, and this License does not cover any rights (other than Database Rights or in contract) in individual Contents contained in the Database. For example, if used on a Database of images (the Contents), this License would not apply to copyright over individual images, which could have their own separate licenses, or one single license covering all of the rights over the images.

3.0 Rights granted

3.1 Subject to the terms and conditions of this License, the Licensor grants to You a worldwide, royalty-free, non-exclusive, terminable (but only under Section 9) license to Use the Database for the duration of any applicable copyright and Database Rights. These rights explicitly include commercial use, and do not exclude any field of endeavour. To the extent possible in the relevant jurisdiction, these rights may be exercised in all media and formats whether now known or created in the future.

The rights granted cover, for example:

a. Extraction and Re-utilisation of the whole or a Substantial part of the Contents;

b. Creation of Derivative Databases;

c. Creation of Collective Databases;

d. Creation of temporary or permanent reproductions by any means and in any form, in whole or in part, including of any Derivative Databases or as a part of Collective Databases; and

e. Distribution, communication, display, lending, making available, or performance to the public by any means and in any form, in whole or in part, including of any Derivative Database or as a part of Collective Databases.

3.2 Compulsory license schemes. For the avoidance of doubt:

a. Non-waivable compulsory license schemes. In those jurisdictions in which the right to collect royalties through any statutory or compulsory licensing scheme cannot be waived, the Licensor reserves the exclusive right to collect such royalties for any exercise by You of the rights granted under this License;

b. Waivable compulsory license schemes. In those jurisdictions in which the right to collect royalties through any statutory or compulsory licensing scheme can be waived, the Licensor waives the exclusive right to collect such royalties for any exercise by You of the rights granted under this License; and,

c. Voluntary license schemes. The Licensor waives the right to collect royalties, whether individually or, in the event that the Licensor is a member of a collecting society that administers voluntary licensing schemes, via that society, from any exercise by You of the rights granted under this License.

3.3 The right to release the Database under different terms, or to stop distributing or making available the Database, is reserved. Note that this Database may be multiple-licensed, and so You may have the choice of using alternative licenses for this Database. Subject to Section 10.4, all other rights not expressly granted by Licensor are reserved.

4.0 Conditions of Use

4.1 The rights granted in Section 3 above are expressly made subject to Your complying with the following conditions of use. These are important conditions of this License, and if You fail to follow them, You will be in material breach of its terms.

4.2 Notices. If You Publicly Convey this Database, any Derivative Database, or the Database as part of a Collective Database, then You must:

a. Do so only under the terms of this License or another license permitted under Section 4.4;

b. Include a copy of this License (or, as applicable, a license permitted under Section 4.4) or its Uniform Resource Identifier (URI) with the Database or Derivative Database, including both in the Database or Derivative Database and in any relevant documentation; and

c. Keep intact any copyright or Database Right notices and notices that refer to this License.

d. If it is not possible to put the required notices in a particular file due to its structure, then You must include the notices in a location (such as a relevant directory) where users would be likely to look for it.

4.3 Notice for using output (Contents). Creating and Using a Produced Work does not require the notice in Section 4.2. However, if you Publicly Use a Produced Work, You must include a notice associated with the Produced Work reasonably calculated to make any Person that uses, views, accesses, interacts with, or is otherwise exposed to the Produced Work aware that Content was obtained from the Database, Derivative Database, or the Database as part of a Collective Database, and that it is available under this License.

a. Example notice. The following text will satisfy notice under Section 4.3:

```
Contains information from DATABASE NAME, which is made available
here under the Open Database License (ODbL).
```

DATABASE NAME should be replaced with the name of the Database and a hyperlink to the URI of the Database. “Open Database License” should contain a hyperlink to the URI of the text of this License. If hyperlinks are not possible, You should include the plain text of the required URI’s with the above notice.

4.4 Share alike.

a. Any Derivative Database that You Publicly Use must be only under the terms of:

i. This License;

ii. A later version of this License similar in spirit to this License; or

iii. A compatible license.

If You license the Derivative Database under one of the licenses mentioned in (iii), You must comply with the terms of that license.

b. For the avoidance of doubt, Extraction or Re-utilisation of the whole or a Substantial part of the Contents into a new database is a Derivative Database and must comply with Section 4.4.

c. Derivative Databases and Produced Works. A Derivative Database is Publicly Used and so must comply with Section 4.4. if a Produced Work created from the Derivative Database is Publicly Used.

d. Share Alike and additional Contents. For the avoidance of doubt, You must not add Contents to Derivative Databases under Section 4.4 a that are incompatible with the rights granted under this License.

e. Compatible licenses. Licensors may authorise a proxy to determine compatible licenses under Section 4.4 a iii. If they do so, the authorised proxy’s public statement of acceptance of a compatible license grants You permission to use the compatible license.

4.5 Limits of Share Alike. The requirements of Section 4.4 do not apply in the following:

a. For the avoidance of doubt, You are not required to license Collective Databases under this License if You incorporate this Database or a Derivative Database in the collection, but this License still applies to this Database or a Derivative Database as a part of the Collective Database;

b. Using this Database, a Derivative Database, or this Database as part of a Collective Database to create a Produced Work does not create a Derivative Database for purposes of Section 4.4; and

c. Use of a Derivative Database internally within an organisation is not to the public and therefore does not fall under the requirements of Section 4.4.

4.6 Access to Derivative Databases. If You Publicly Use a Derivative Database or a Produced Work from a Derivative Database, You must also offer to recipients of the Derivative Database or Produced Work a copy in a machine readable form of:

a. The entire Derivative Database; or

b. A file containing all of the alterations made to the Database or the method of making the alterations to the Database (such as an algorithm), including any additional Contents, that make up all the differences between the Database and the Derivative Database.

The Derivative Database (under a.) or alteration file (under b.) must be available at no more than a reasonable production cost for physical distributions and free of charge if distributed over the internet.

4.7 Technological measures and additional terms

a. This License does not allow You to impose (except subject to Section 4.7 b.) any terms or any technological measures on the Database, a Derivative Database, or the whole or a Substantial part of the Contents that alter or restrict the terms of this License, or any rights granted under it, or have the effect or intent of restricting the ability of any person to exercise those rights.

b. Parallel distribution. You may impose terms or technological measures on the Database, a Derivative Database, or the whole or a Substantial part of the Contents (a “Restricted Database”) in contravention of Section 4.74 a. only if You also make a copy of the Database or a Derivative Database available to the recipient of the Restricted Database:

i. That is available without additional fee;

ii. That is available in a medium that does not alter or restrict the terms of this License, or any rights granted under it, or have the effect or intent of restricting the ability of any person to exercise those rights (an “Unrestricted Database”); and

iii. The Unrestricted Database is at least as accessible to the recipient as a practical matter as the Restricted Database.

c. For the avoidance of doubt, You may place this Database or a Derivative Database in an authenticated environment, behind a password, or within a similar access control scheme provided that You do not alter or restrict the terms of this License or any rights granted under it or have the effect or intent of restricting the ability of any person to exercise those rights.

4.8 Licensing of others. You may not sublicense the Database. Each time You communicate the Database, the whole or Substantial part of the Contents, or any Derivative Database to anyone else in any way, the Licensor offers to the recipient a license to the Database on the same terms and conditions as this License. You are not responsible for enforcing compliance by third parties with this License, but You may enforce any rights that You have over a Derivative Database. You are solely responsible for any modifications of a Derivative Database made by You or another Person at Your direction. You may not impose any further restrictions on the exercise of the rights granted or affirmed under this License.

5.0 Moral rights

5.1 Moral rights. This section covers moral rights, including any rights to be identified as the author of the Database or to object to treatment that would otherwise prejudice the author's honour and reputation, or any other derogatory treatment:

a. For jurisdictions allowing waiver of moral rights, Licensor waives all moral rights that Licensor may have in the Database to the fullest extent possible by the law of the relevant jurisdiction under Section 10.4;

b. If waiver of moral rights under Section 5.1 a in the relevant jurisdiction is not possible, Licensor agrees not to assert any moral rights over the Database and waives all claims in moral rights to the fullest extent possible by the law of the relevant jurisdiction under Section 10.4; and

c. For jurisdictions not allowing waiver or an agreement not to assert moral rights under Section 5.1 a and b, the author may retain their moral rights over certain aspects of the Database.

Please note that some jurisdictions do not allow for the waiver of moral rights, and so moral rights may still subsist over the Database in some jurisdictions.

6.0 Fair dealing, Database exceptions, and other rights not affected

6.1 This License does not affect any rights that You or anyone else may independently have under any applicable law to make any use of this Database, including without limitation:

a. Exceptions to the Database Right including: Extraction of Contents from non-electronic Databases for private purposes, Extraction for purposes of illustration for teaching or scientific research, and

Extraction or Re-utilisation for public security or an administrative or judicial procedure.

b. Fair dealing, fair use, or any other legally recognised limitation or exception to infringement of copyright or other applicable laws.

6.2 This License does not affect any rights of lawful users to Extract and Re-utilise insubstantial parts of the Contents, evaluated quantitatively or qualitatively, for any purposes whatsoever, including creating a Derivative Database (subject to other rights over the Contents, see Section 2.4). The repeated and systematic Extraction or Re-utilisation of insubstantial parts of the Contents may however amount to the Extraction or Re-utilisation of a Substantial part of the Contents.

7.0 Warranties and Disclaimer

7.1 The Database is licensed by the Licensor "as is" and without any warranty of any kind, either express, implied, or arising by statute, custom, course of dealing, or trade usage. Licensor specifically disclaims any and all implied warranties or conditions of title, non-infringement, accuracy or completeness, the presence or absence of errors, fitness for a particular purpose, merchantability, or otherwise. Some jurisdictions do not allow the exclusion of implied warranties, so this exclusion may not apply to You.

8.0 Limitation of liability

8.1 Subject to any liability that may not be excluded or limited by law, the Licensor is not liable for, and expressly excludes, all liability for loss or damage however and whenever caused to anyone by any use under this License, whether by You or by anyone else, and whether caused by any fault on the part of the Licensor or not. This exclusion of liability includes, but is not limited to, any special, incidental, consequential, punitive, or exemplary damages such as loss of revenue, data, anticipated profits, and lost business. This exclusion applies even if the Licensor has been advised of the possibility of such damages.

8.2 If liability may not be excluded by law, it is limited to actual and direct financial loss to the extent it is caused by proved negligence on the part of the Licensor.

9.0 Termination of Your rights under this License

9.1 Any breach by You of the terms and conditions of this License automatically terminates this License with immediate effect and without notice to You. For the avoidance of doubt, Persons who have received the Database, the whole or a Substantial part of the Contents, Derivative Databases, or the Database as part of a Collective Database from You under this License will not have their licenses terminated provided their use is in full compliance with this License or a license granted under Section 4.8 of this License. Sections 1, 2, 7, 8, 9 and 10 will survive any termination of this License.

9.2 If You are not in breach of the terms of this License, the Licensor will not terminate Your rights under it.

9.3 Unless terminated under Section 9.1, this License is granted to You for the duration of applicable rights in the Database.

9.4 Reinstatement of rights. If you cease any breach of the terms and conditions of this License, then your full rights under this License will be reinstated:

a. Provisionally and subject to permanent termination until the 60th day after cessation of breach;

b. Permanently on the 60th day after cessation of breach unless otherwise reasonably notified by the Licensor; or

c. Permanently if reasonably notified by the Licensor of the violation, this is the first time You have received notice of violation of this License from the Licensor, and You cure the violation prior to 30 days after your receipt of the notice.

Persons subject to permanent termination of rights are not eligible to be a recipient and receive a license under Section 4.8.

9.5 Notwithstanding the above, Licensor reserves the right to release the Database under different license terms or to stop distributing or making available the Database. Releasing the Database under different license terms or stopping the distribution of the Database will not withdraw this License (or any other license that has been, or is required to be, granted under the terms of this License), and this License will continue in full force and effect unless terminated as stated above.

10.0 General

10.1 If any provision of this License is held to be invalid or unenforceable, that must not affect the validity or enforceability of the remainder of the terms and conditions of this License and each remaining provision of this License shall be valid and enforced to the fullest extent permitted by law.

10.2 This License is the entire agreement between the parties with respect to the rights granted here over the Database. It replaces any earlier understandings, agreements or representations with respect to the Database.

10.3 If You are in breach of the terms of this License, You will not be entitled to rely on the terms of this License or to complain of any breach by the Licensor.

10.4 Choice of law. This License takes effect in and will be governed by the laws of the relevant jurisdiction in which the License terms are sought to be enforced. If the standard suite of rights granted under applicable copyright law and Database Rights in the relevant jurisdiction includes additional rights not granted under this License, these additional rights are granted in this License in order to meet the terms of this License.

MORE INFORMATION

- Introduction to Open Data
- Open Definition for Data
- Quick guide to making data open
- Open Data Handbook

- **Slovenia**: Contains data from the Surveying and Mapping Authority and Ministry of Agriculture, Forestry and Food (public information of Slovenia).
- **South Africa**: Contains data sourced from Chief Directorate: National Geo-Spatial Information, State copyright reserved.
- **United Kingdom**: Contains Ordnance Survey data © Crown copyright and database right 2010-12.

For further details of these, and other sources that have been used to help improve OpenStreetMap, please see the Contributors page on the OpenStreetMap Wiki.

Inclusion of data in OpenStreetMap does not imply that the original data provider endorses OpenStreetMap, provides any warranty, or accepts any liability.

Copyright infringement

OSM contributors are reminded never to add data from any copyrighted sources (e.g. Google Maps or printed maps) without explicit permission from the copyright holders.

If you believe that copyrighted material has been inappropriately added to the OpenStreetMap database or this site, please refer to our takedown procedure or file directly at our on-line filing page.

Trademarks

OpenStreetMap, the magnifying glass logo and State of the Map are registered trademarks of the OpenStreetMap Foundation. If you have questions about your use of the marks, please send your questions to the Licence Working Group.
